# Supplementary material for: An Optimized SP3 Sample Processing Workflow for In-Depth and Reproducible Phosphoproteomics
Source: J Proteome Res. 2025 Jul 17;24(8):4300–8. doi: 10.1021/acs.jproteome.5c00220 (PMC12322947; doi:10.1021/acs.jproteome.5c00220)
Supplement: Supplementary file 1 [file pr5c00220_si_001.pdf]

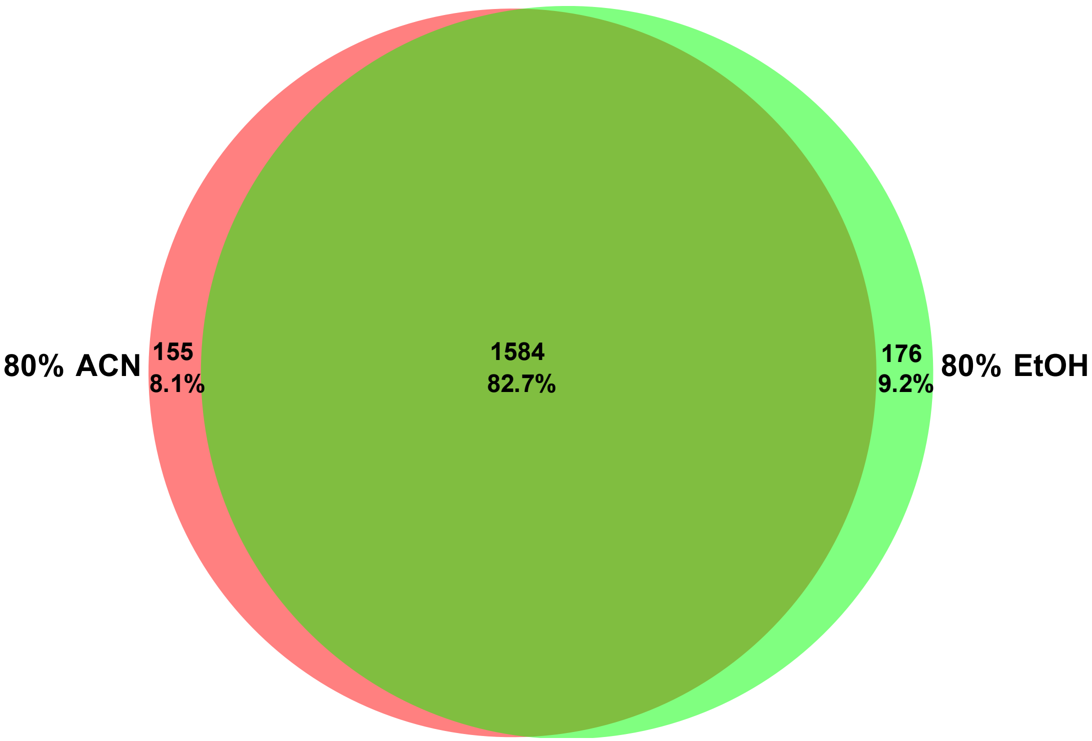

Supp. Figure 1: Protein level differences between precipitation solvents. *Lysate without protease inhibitors and phosSTOP were precipitated with different organic solvents. Venn diagram of protein Identifications observed in ≥2 replicates.*
