# Supplementary material for: An Optimized SP3 Sample Processing Workflow for In-Depth and Reproducible Phosphoproteomics
Source: J Proteome Res. 2025 Jul 17;24(8):4300–8. doi: 10.1021/acs.jproteome.5c00220 (PMC12322947; doi:10.1021/acs.jproteome.5c00220)
Supplement: Supplementary file 2 [file pr5c00220_si_002.pdf]

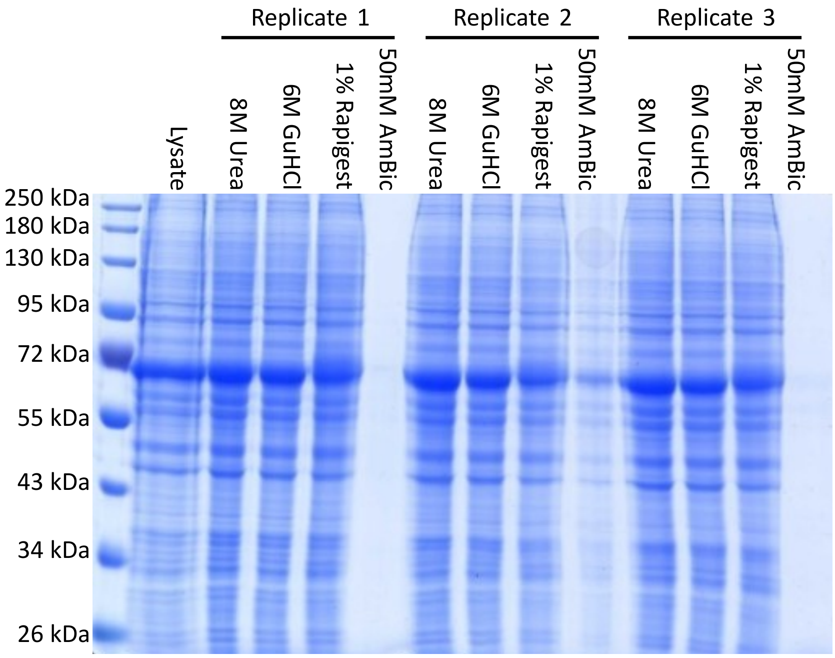

Supp. Figure 2: Protein elution strategies from SP3 beads*Coomassie gel of lysate (without protease inhibitors and phosSTOP) precipitated with 80% EtOH onto SP3 beads and eluted using stated solution, alongside an equal loading of non-precipitated lysate.*
