# Supplementary material for: An Optimized SP3 Sample Processing Workflow for In-Depth and Reproducible Phosphoproteomics
Source: J Proteome Res. 2025 Jul 17;24(8):4300–8. doi: 10.1021/acs.jproteome.5c00220 (PMC12322947; doi:10.1021/acs.jproteome.5c00220)
Supplement: Supplementary file 3 [file pr5c00220_si_003.pdf]

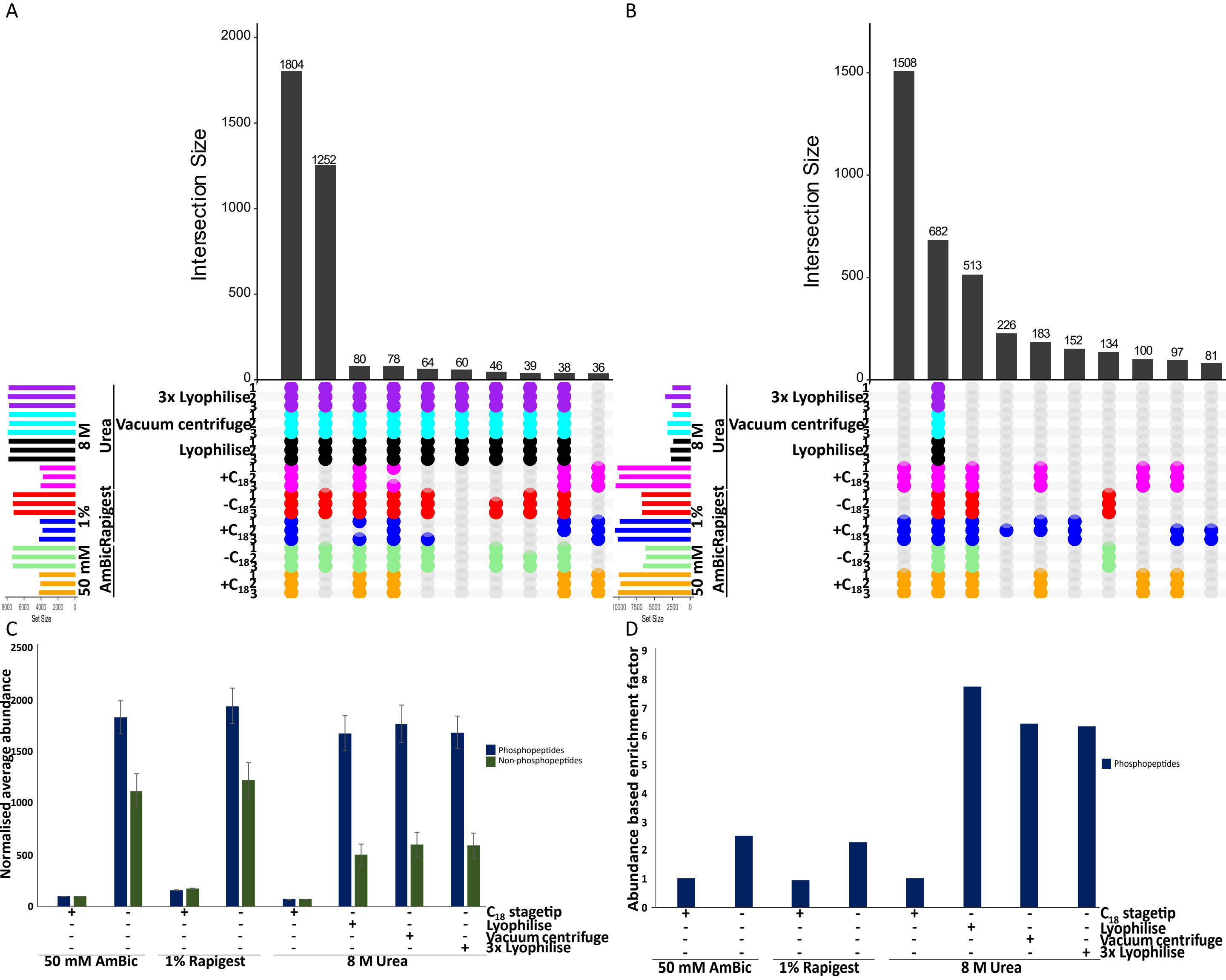

Supp. Figure 3: Evaporative removal for urea elution strategies increase phosphopeptide identifications and enrichment efficiency. *SP3 beads were post digest washed in stated solution, and subject to either ±C18 SPE clean-up or, for urea removal, evaporative strategies prior to TiO_2 phosphopeptide enrichment. A total of 10,508 phospho- and 17,501 non-phospho- peptides were identified post enrichment. A) UpSetPlot of phosphopeptides identified across conditions. B) UpSetPlot of non-phosphopeptides identified across conditions. C) Normalized average abundance of phospho- and non-phospho- peptides observed in all replicates of each condition versus 50 mM AmBiC +C18 (1804 and 682 respectively). D) Abundance based enrichment factor. Calculation (Average abundance of phosphopeptides / average abundance of non-phosphopeptides) post removal of the Top 20 differentially abundant non-phosphopeptides.*
