# Supplementary material for: An Optimized SP3 Sample Processing Workflow for In-Depth and Reproducible Phosphoproteomics
Source: J Proteome Res. 2025 Jul 17;24(8):4300–8. doi: 10.1021/acs.jproteome.5c00220 (PMC12322947; doi:10.1021/acs.jproteome.5c00220)
Supplement: Supplementary file 4 [file pr5c00220_si_004.pdf]

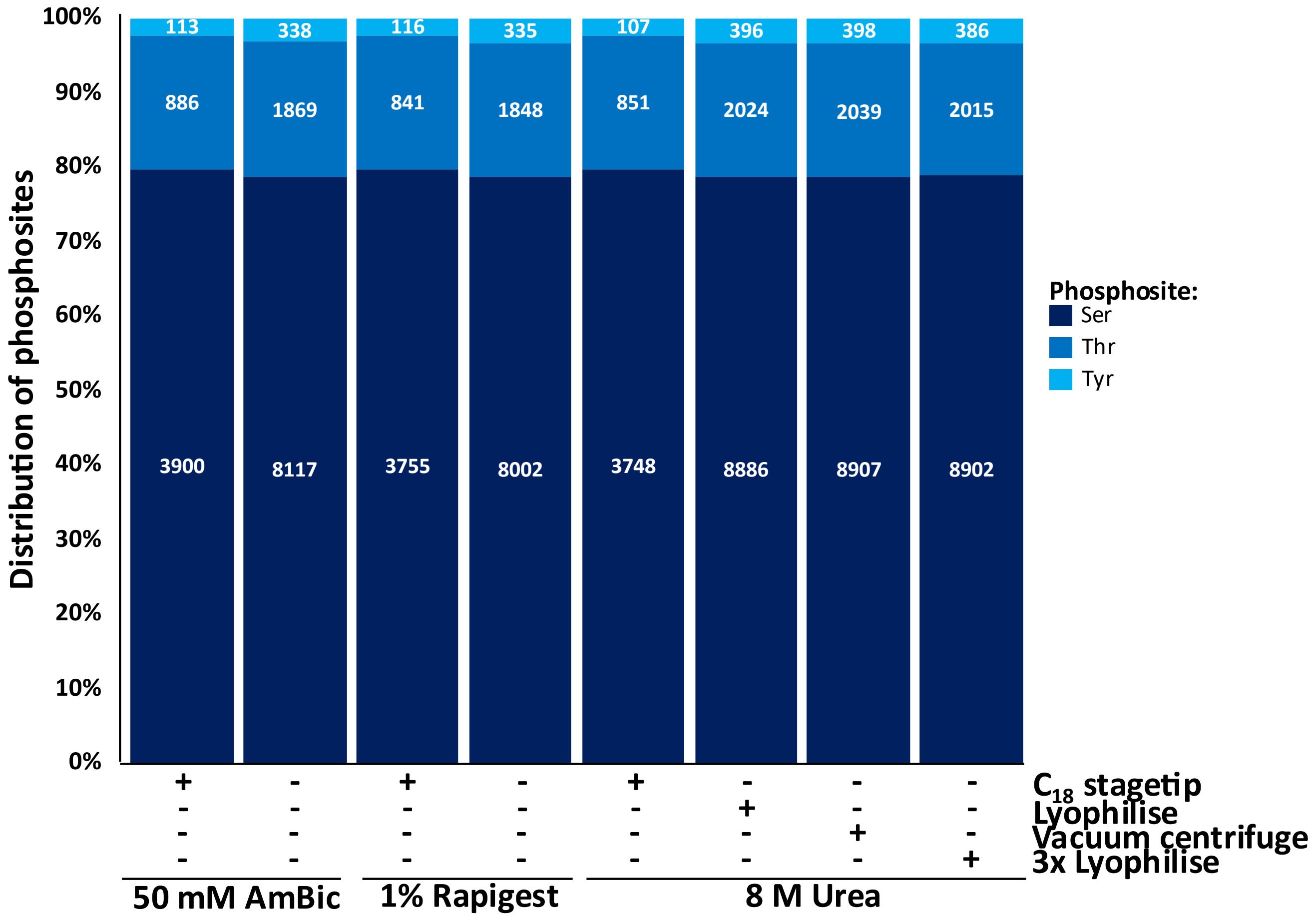

Supp. Figure 4: Phosphosite distribution remains unchanged during optimization of the processing pipeline
